# Feeding regimen modulates zebrafish behavior

**DOI:** 10.7717/peerj.5343

**Published:** 2018-08-03

**Authors:** Fernanda S. Dametto, Débora Fior, Renan Idalencio, João Gabriel S. Rosa, Michele Fagundes, Alessandra Marqueze, Rodrigo E. Barreto, Angelo Piato, Leonardo J.G. Barcellos

**Affiliations:** 1 Programa de Pós-Graduação em Bioexperimentação, Universidade de Passo Fundo (UPF), Passo Fundo, RS, Brazil; 2 Programa de Pós-Graduação em Farmacologia, Universidade Federal de Santa Maria (UFSM), Santa Maria, RS, Brazil; 3 Faculdade de Agronomia e Medicina Veterinária, Universidade de Passo Fundo, Passo Fundo, Brazil; 4 Curso de Medicina Veterinária, UniSociesc, Blumenau, Brasil; 5 Programa de Pós-Graduação em Ciências Ambientais, Universidade de Passo Fundo, Passo Fundo, RS, Brasil; 6 Programa de Pós-Graduação em Avaliação de Impactos Ambientais em Mineração, Unilasalle, Canoas, Brasil; 7 Departmento de Fisiologia, Instituto de Biociências, UNESP, CAUNESP, Botucatu, São Paulo, Brasil; 8 Programa de Pós-Graduação em Farmacologia e Terapêutica, Universidade Federal do Rio Grande do Sul, Porto Alegre, Brazil

**Keywords:** Anxiety, Novel tank test, *Danio rerio*, Energetic metabolism, Feeding frequency

## Abstract

Here we show that the feeding regimen modulates zebrafish (*Danio rerio*) behavior. With regard to the time elapsed between feeding and behavioral evaluation, fish fed 3 h before behavioral evaluation in the novel tank test (NTT) showed decreased activity and a trend toward an anxiolytic reaction (increased use of the upper section of the aquarium) in comparison to fish fed 0.5, 6, 12, 24 or 48 h before testing, although differences were not statistically significant for all comparisons. Activity and use of the upper section of the aquarium did not differ significantly among the other treatments. Regarding feeding frequency, fish fed once a day showed higher anxiety-like behavior (decreased use of the upper section of the aquarium) in comparison to fish fed twice a day, but feeding four or six times per day or only every second day did not result in differences from feeding twice a day. Feeding frequency had no effect on activity level. Metabolically, fish fed once a day presented decreased levels of glucose and glycogen and increased lactate when compared to the regular feeding (fish fed twice a day), suggesting that feeding regimen may modulate carbohydrate metabolism. Mechanistically, we suggest that the metabolic changes caused by the feeding regimen may induce behavioral changes. Our results suggest that the high variability of the results among different laboratories might be related to different feeding protocols. Therefore, if issues pertaining to the feeding regimen are not considered during experiments with zebrafish, erroneous interpretations of datasets may occur.

## Introduction

The zebrafish (*Danio rerio*) is widely used as an experimental animal model in a variety of research fields in biomedical research, as its genome has high homology with the human genome (Barbazuk et al., 2000). Moreover, the zebrafish has straightforward breeding conditions, initial transparency, rapid development (Spence et al., 2008), and easy husbandry (Lawrence, 2007). Zebrafish are also suited for behavioral screenings (Egan et al., 2009), representing a cost-effective and efficient alternative to rodents (Barbazuk et al., 2000; Piato et al., 2011). In this context, the zebrafish has been used in many specific research fields such as genetics, neuroscience, and pharmacology (Norton & Bally-Cuif, 2010; Tierney, 2011), to evaluate its behavioral repertoire for understanding many phenomena, such as anxiety and stress responses, indicating its utility as a robust and quantifiable research tool (Stewart et al., 2011).

Despite the well-known zebrafish behavioral repertoire (Kalueff et al., 2013), discrepancies and variability in research outcomes need to be considered. Some sources of variability have already been studied, such as fish personality traits (Wilson et al., 1994; Réale et al., 2007), immune status (Kirsten et al., 2018), sex (Rambo et al., 2016; Reolon et al., 2018), strains (Canzian et al., 2017; Rosa et al., 2018), previous predatory experiences (Barcellos et al., 2010; Abreu et al., 2018), and exposure to agrichemicals (Santos da Rosa et al., 2017) and drug residues (Barcellos et al., 2016; Abreu et al., 2016; Kalichak et al., 2016). These possible interfering factors may impair the reproducibility of the experiments and the accuracy of the results.

Studies focused on nutritional issues such as nutrient balance are frequent in rats (Boitard et al., 2012), humans (Agarwal et al., 2015) and in the zebrafish (O’Brine et al., 2015; Forsatkar et al., 2017; McDougall et al., 2017). However, studies on feeding regimen are scarce and focused on production aspects such as fish health in salmon (Frenzl et al., 2014; Jones et al., 2018) and growth performance in Korean rockfish (*Sebastes schlegeli*) (Lee, Hwang & Cho, 2000), yellowtail flounder (*Limanda ferruginea*) (Dwyer et al., 2002) and Atlantic salmon (*Salmo salar*) (Shi et al., 2017).

There have been experimental studies on the relationship between feeding regimen (frequency and rate) and swimming performance and metabolism in fishes (Thorarensen & Farrell, 2006; Dong et al., 2015; Nie & Fu, 2017; Nie, Cao & Fu, 2017). However, to the best of our knowledge, there have been no experimental studies on behavioral effects of the frequency of feeding or the time between last feeding and behavioral testing in zebrafish, especially with regard to anxiety-like behavior.

In the present study, we investigated the feeding regimen of laboratory zebrafish stocks as a candidate source for variation in results from different laboratories around the world. Specifically, we opted to manipulate two variables in feeding regimens that can be easily altered within research facilities and that may affect behavioral outcomes: feeding frequency and time between the last feeding and behavioral testing. Because the existent literature relates feeding regimen to swimming performance and metabolism (Thorarensen & Farrell, 2006; Nie & Fu, 2017; Nie, Cao & Fu, 2017), we also measured metabolic parameters like glucose, glycogen and lactate content as a possible causal mechanism of resulting behavior changes.

To test the hypothesis that feeding regimen affects fish behavior, we measured locomotor and anxiety-related behavioral outcomes. For this purpose, we used the novel tank test (NTT). NTT is an easy and reliable test based on geotaxis, the innate escape diving behavior of fish in novel environments (Kysil et al., 2017). This test represents a conceptual analog of the rodent open field since NTT evokes motivational conflicts between diving behavior (a protective reaction) and subsequent vertical exploration. NTT targets the phenotypic domains of exploration and locomotion. Regarding anxiety-related endpoints, NTT assesses several parameters such as the time spent in the upper/bottom zone, latency to enter the top, and number of crossings between the zones (Kysil et al., 2017).

## Materials and Methods

### Animals and housing conditions

A population of 180 mixed-sex adult wild-type zebrafish (sex ratio 1:1) of the short-fin (SF) phenotype, weighing 0.3–0.5 g, from a single brood of heterogeneous breeding stock at the Universidade de Passo Fundo, Brazil, was kept in 100-L plastic tanks with constant aeration and equipped with biological filtering under a natural photoperiod (14 h light/10 h dark). During the pre-experimental period and in the experiments, fish were fed with a commercial flaked food (Alcon Basic ©, humidity 10%, raw protein 45%, fat extract 5%). Water temperature was maintained at 28 ± 2 °C, pH 7.0 ± 0.2, dissolved oxygen at 6.1 ± 0.2 mg/L, total ammonia at <0.01 mg/L, total hardness at 6 mg/L, and alkalinity at 22 mg/L CaCO_3_.

### Ethics

The study was approved by the Ethics Commission for Animal Use (CEUA) at Universidade de Passo Fundo, UPF, Passo Fundo, RS, Brazil (Protocol 32/2016) and fully complied with the guidelines of Conselho Nacional de Controle de Experimentação Animal (CONCEA).

### Specific procedures

#### Experiment 1

To evaluate the effect of the interval between feeding and behavioral testing, we captured 72 zebrafish haphazardly from different aquaria within a 48-aquaria recirculating water system, and distributed among 24 glass aquaria (3.9 L, 20 × 15 × 15 cm L × W × H, 3 fish/aquarium), with four aquaria for each of the six experimental groups. Fish were weighed to determine the amount of food provided. Fish were gently captured, placed on a small piece of filter paper, and rapidly weighed using a digital balance. The entire procedure took less than 30 s (fish weights are provided in the Supplemental Information 1). During 10 days of acclimation, fish were fed using the regular feeding routine of the laboratory (twice a day), at a rate of 3% body weight per fish per day. All remaining food residue was removed from the water surface after 5 min. Observations during the feeding period indicated that all fish ate similar amounts of food. After 10 days of acclimation, we varied the interval between the last feeding and presentation of fish to the test (0.5, 3, 6, 12, 24 h and 48 h). Caution was taken to ensure that the interval between feeding and testing was the same for all fish within each group. In this experiment, the last feeding was at 06:00 and NTT testing was conducted from 06:30 to 18:00 h for 3 consecutive days (first day for the intervals from 0.5 to 12 h, second day at 06:00 h for the 24-h interval, and third day at 06:00 h for the 48-h interval).

#### Experiment 2

**Figure 1:**
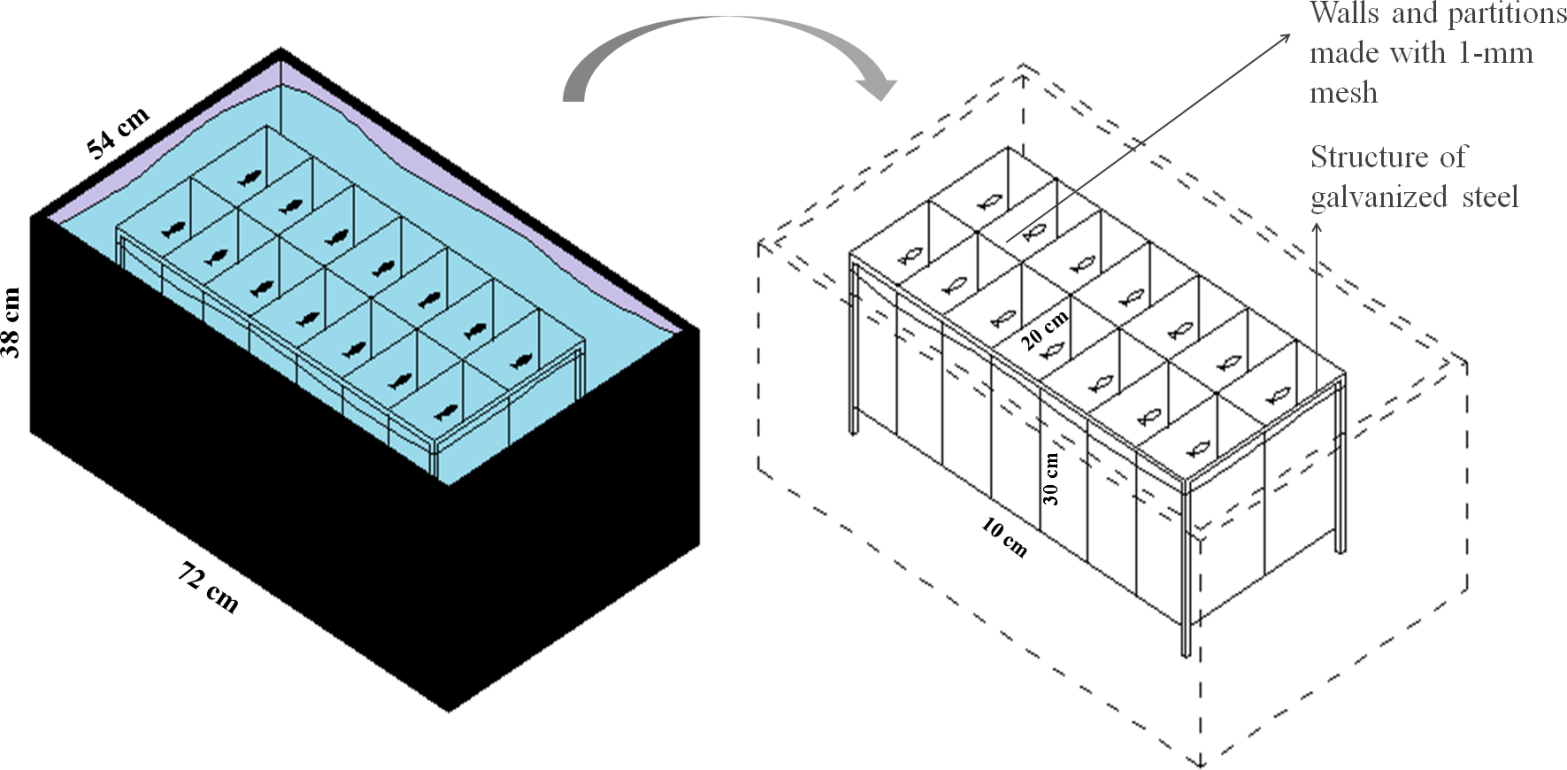
Schematic representation of the isolation apparatus used in Experiment 2.

To evaluate the effect of feeding frequency on behavioral testing, we weighed and distributed 70 zebrafish, haphazardly captured from a 48-aquaria recirculating water system, among five plastic tanks (147 L, 72 × 54 × 38 cm, L × W × H). We placed 14 fish into each plastic tank for a total of five experimental groups (*n* = 14). Within each tank, we installed an apparatus for physical isolation and identification of each individual while allowing chemical communication and visual contact (Fig. 1). Each chamber measured 10 × 20 × 30 cm (L × W × H), with a volume of 6 L, and was delimited by a 1-mm plastic mesh. Fish from the five treatment groups were haphazardly allocated to the plastic tanks and were individually identified to ensure that each tank was allocated fish from each group. All fish were weighed to determine the amount of food as described in Experiment 1 (fish weight is provided in Supplemental Information 1). In all five groups, fish were fed at a rate of 3% of body weight per day. The total amount of food was divided according to the different feeding frequencies as depicted in Table 1. We considered feeding twice a day as regular feeding based on the routine of our laboratory. All eventual remaining food residue was removed from the water surface after 5 min.

**Table 1:** Feeding frequency and time in Experiment 2.

Group	Frequency	Feeding time
1	6 times a day	08:00, 10:00, 12:00, 15:00, 17:00 and 19:00
2	4 times a day	08:00, 12:00, 15:00 and 19:00
3	Twice a day (regular feeding)	08:00 and 19:00
4	Once a day	08:00
5	Once every two days	08:00

 After 15 days of the different feeding regimens, we submitted fish individually to the NTT. For all treatments, care was taken to ensure a time interval of 12 h between the last feeding and behavioral testing. Considering chronobiological factors, all tests were conducted from 06:00 to 10:00 h. After NTT, we weighed and measured all fish. Fish were then sacrificed and stored in liquid nitrogen for biochemical analyses.

#### Novel tank test

We used rectangular glass aquaria (24 × 8 × 20 cm, L × W × H) (Mocelin et al., 2015) filled with water from the same source and with the same characteristics of the water of experimental aquaria. Fish were filmed for 7 min using a Logitech HD Webcam C525 camera positioned on a tripod in front of the test aquarium. To avoid interference by human activity, the operator exited the experimental room after the fish were released into the NTT. For video analysis, we did not consider the initial 60-s interval, which was a period to allow fish adjustment to the stress of transfer and the novel conditions. The videos were then analyzed offline using the ANY-maze^^®^^ software, in which the test tank was divided into top, middle, and bottom zones. We scored the following behavioral parameters:: total distance traveled (m), number of rotations (also called circling, a 360° change in orientation in the horizontal plane), number of crossings between zones (considering the full body), time spent in the top (s), and the latency to enter in the top zone (considering the length of time elapsed before animal entry in the top zone for the first time [s]).

#### Metabolism

The metabolism variables were determined only in fish from Experiment 2. Whole body biochemical analyses were performed due to the small size of our study fish. The whole body glucose level was determined using a glucose oxidase kit (Labtest, MG, Brazil). Glycogen content was determined by the Van Handel method (Van Handel, 1965). Lactate concentration was determined using a commercial kit (Kit Vis Interteck/Katal) (Da Rosa et al., 2017).

#### Statistical analyses

In Experiment 1, to meet the assumptions of the analysis of variance with regard to the independence of variables, we used the mean value of each aquarium, resulting in four true replicates of each treatment. The different intervals between the last feeding and the NTT were compared using one-way ANOVA followed by a post-hoc Tukey’s multiple range test or a Kruskal–Wallis test followed by a post-hoc Dunn’s test, depending on normality of the data (assessed by the Kolmogorov–Smirnov test). In Experiment 2, we compared the different feeding frequencies against the regular frequency (twice a day), using a one-way ANOVA followed by a post-hoc Dunnet’s test or a Kruskal–Wallis test followed by a post-hoc Dunn’s test, depending on normality of the data. The alpha level was set at 0.05 in Experiment 1 and set at a more conservative value of 0.01 in Experiment 2, because fish in the five treatments had been allocated to the five tanks (i.e., each tank contained fish from the five groups). This strategy allowed a relative but not complete independence of variables. In addition, using a conservative alpha level we reduced the chance of type I error.

## Results

### Experiment 1

**Figure 2:**
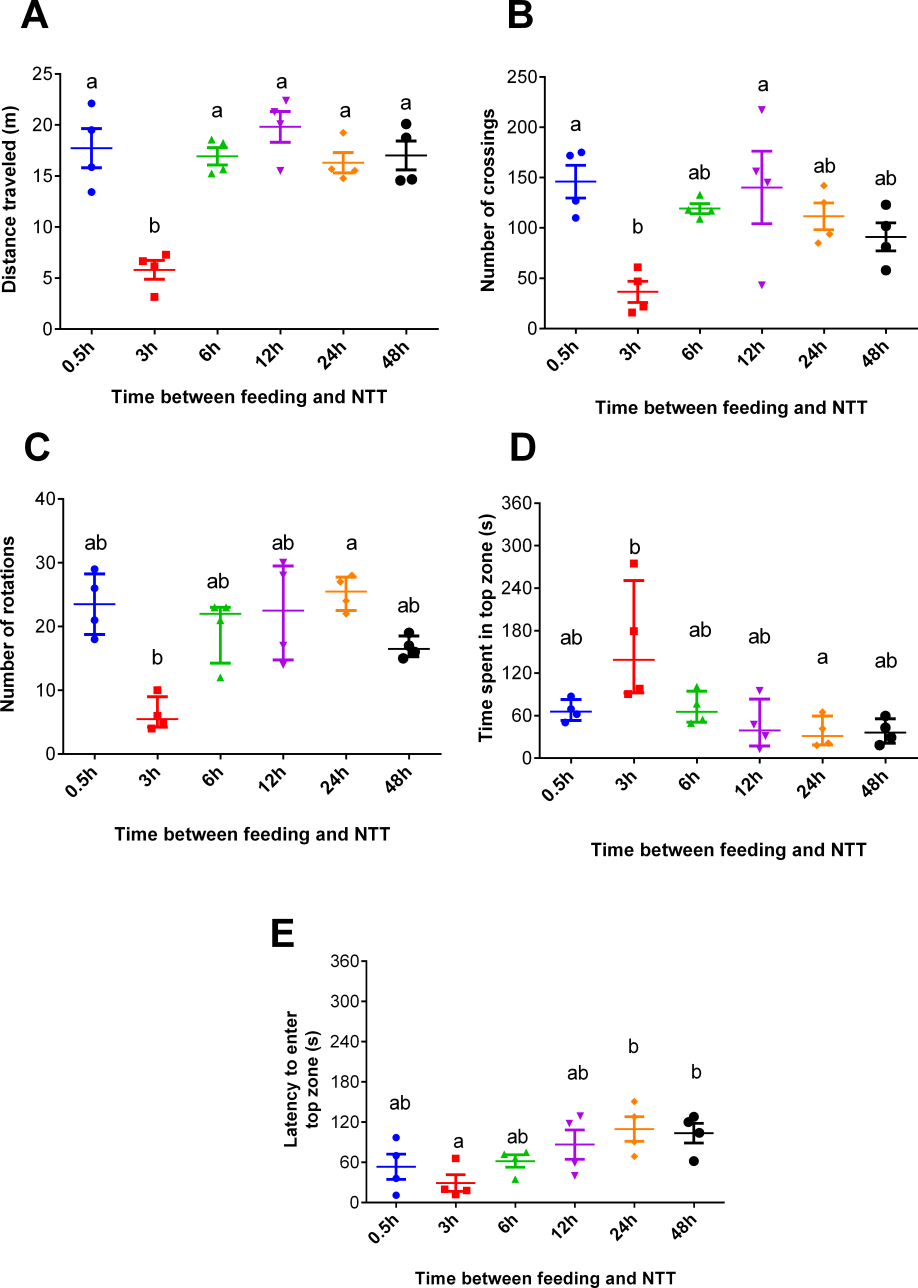
Behavioral variables of zebrafish fed 0.5, 3, 6, 12, 24, and 48 h before a novel tank test. (A) total distance traveled per individual (m), (B) total number of crossings per individual, (C) total number of rotations per individual, (D) total time spent in the top zone per individual (s), and (E) latency to enter the top zone (s) during the 6-min test. Individual points represent means of three individuals from each of four aquaria. In (A, B, and E), horizontal lines represent the mean ± S.E., and different letters indicate significant differences (*p* < 0.05) between treatments by Tukey’s multiple range test. In (C and D), horizontal lines represent the median and interquartile range, and different letters represent significant differences (*p* < 0.05) by Dunn’s test.

Fish fed 3 h before the NTT traveled less than those tested at all other times (*F*_5,18_ = 13.93, *p* < 0.0001, Fig. 2A). Regarding crossing number, fish fed 3 h before the NTT crossed less than fish fed 0.5 and 12 h before the NTT (*F*_5,18_ = 4.641, *p* = 0.0068, Fig. 2B). Fish fed 3 h before the NTT performed a smaller number of rotations than fish fed 24 h before the NTT (*F*_5,18_ = 13.91, *p* = 0.0162, Fig. 2C), although differences were not statistically significant for all comparisons. Fish fed 3 h before the NTT spent more time in the top zone than fish fed 24 h before NTT (*F*_5,18_ = 13.46, *p* = 0.0194, Fig. 2D) and had a shorter latency to enter the top zone compared to fish fed 24 and 48 h before NTT (*F*_5,18_ = 3.612, *p* = 0.0194, Fig. 2E). The use and the latency to enter the upper section of the aquarium did not differ significantly among the other treatments. Please refer to the supplementary information for additional statistical details.

### Experiment 2

**Figure 3:**
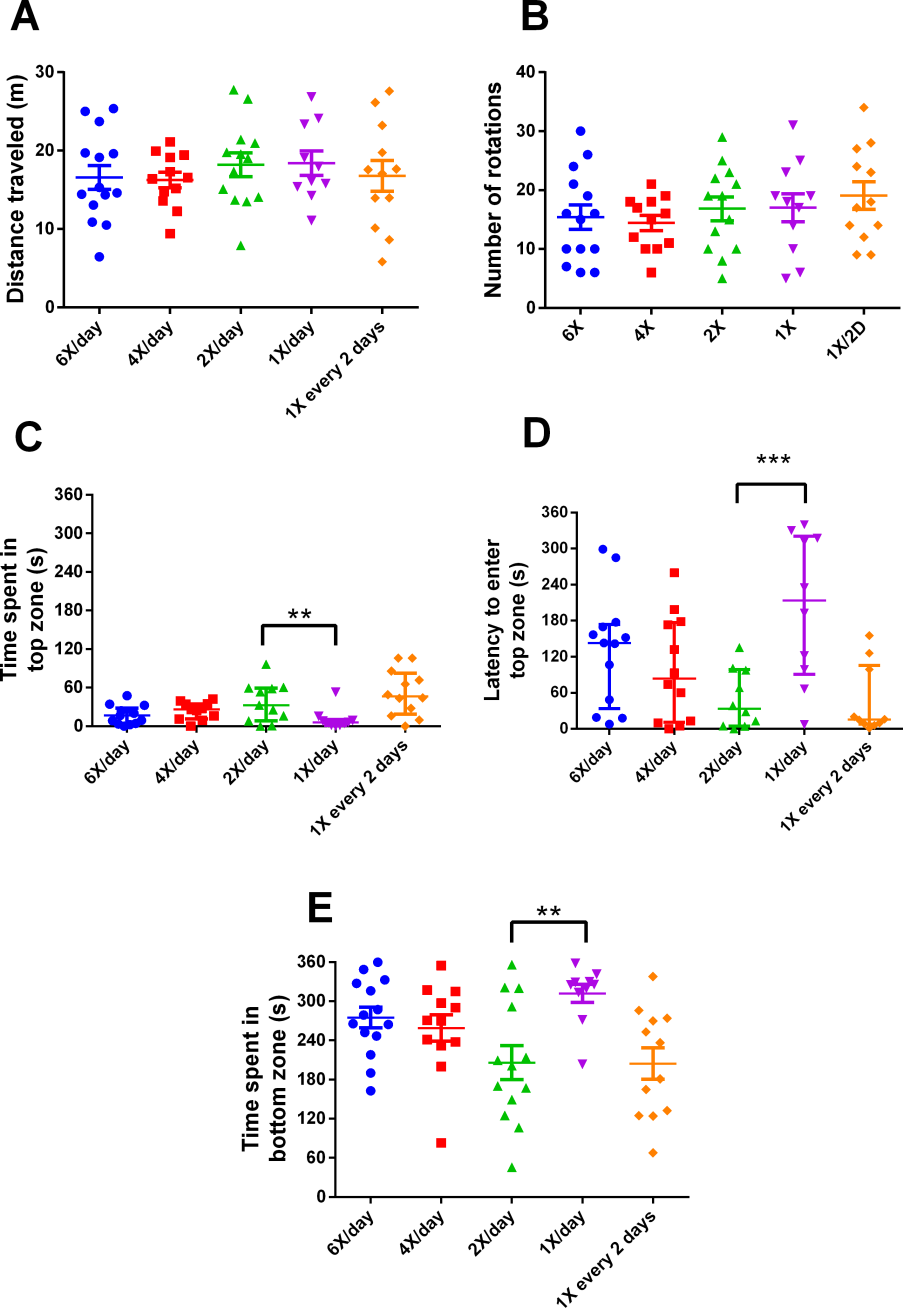
Behavioral variables of zebrafish in the novel tank test in different feeding frequencies for 15 days. (A) total distance traveled per individual (m), (B) total number of rotations per individual, (C) time spent in the top zone per individual (s), (D) latency to enter the top zone (s), and (E) time spent in the bottom zone (s) during the 6-min test. In (A, B and E), horizontal lines represent the mean ± S.E., and asterisks over the brackets indicate statistical difference compared to twice a day regular feeding frequency by one-way ANOVA followed by Dunnet’s test. In (C and D), horizontal lines represent the median and interquartile range and asterisks over the brackets indicate statistical difference compared to twice a day regular feeding frequency by Kruskal–Wallis followed by Dunn’s test. (** *p* < 0.01 and *** *p* < 0.001).

**Figure 4:**
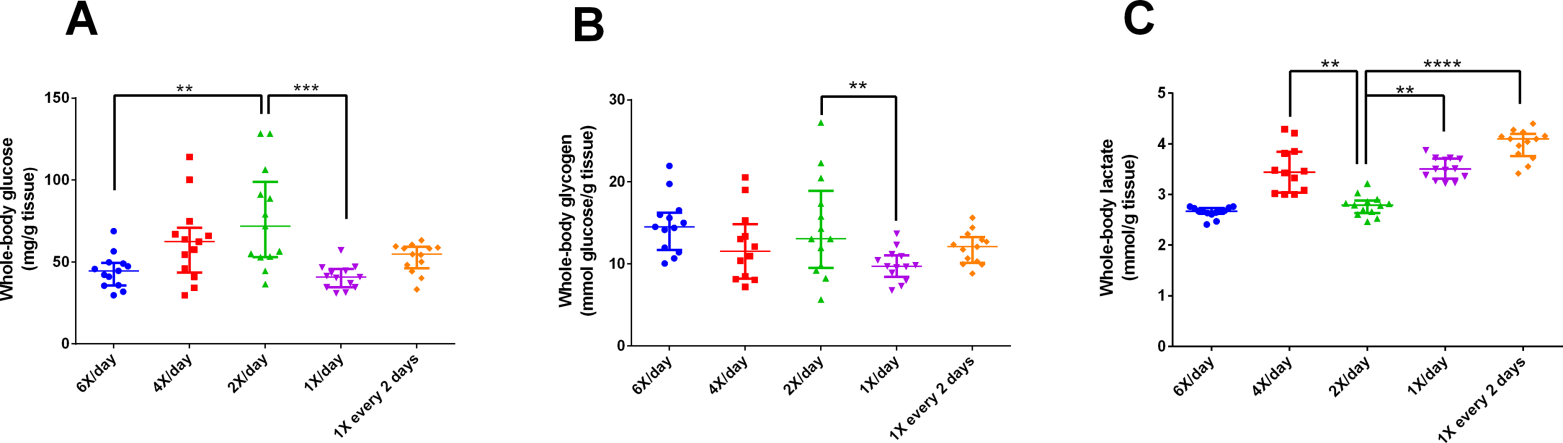
Intermediary metabolic variables of zebrafish fed at different feeding frequencies for 15 days. (A) whole-body glucose (mg/g tissue), (B) whole-body glycogen (mmol glucose/g tissue), and (C) whole-body lactate (mmol/g tissue). Horizontal lines represent the median and interquartile range and asterisks over the brackets indicate statistical difference compared to twice a day regular feeding frequency by Kruskal–Wallis followed by Dunn’s test. (**p < 0.01, ***p < 0.001 and *p* < 0.0001).

After 15 days of different feeding frequencies in individually housed zebrafish, individuals fed once a day (24 h frequency) presented anxiety-like behavior compared with those subjected to regular feeding frequency (twice a day). This was evident from the decreased time spent in the top (Kruskal–Wallis statistics *H* = 14.65, *p* = 0.0055, *df* = 4,56, Fig. 3C), increased latency to enter the top zone (*H* = 14.72, *p* = 0.0053, *df* = 4,54, Fig. 3D) and increased time spent in the bottom zone (*F*_4,56_ = 4.641, *p* = 0.0027, Fig. 3F). However, feeding 4 or 6 times per day or only every second day did not result in differences from feeding twice a day. There were no treatment effects on locomotor variables (Figs. 3A,  3B). Regarding metabolism, fish fed six times a day and those fed once a day had reduced whole-body glucose levels in comparison with fish fed twice a day (*H* = 20.97, *p* = 0.0003, *df* = 4,64, Fig. 4A). Fish fed once a day were the only treatment to show a reduction in whole-body glycogen levels in comparison with fish fed twice a day (*H* = 14.58, *p* = 0.0057, *df* = 4,64, Fig. 4B). Fish fed four times and once a day, as well as fish, fed every 48 h had increased levels of whole-body lactate in comparison with fish fed twice a day (*H* = 49.61, *p* < 0.0001, *df* = 4,64, Fig. 4C). Although there were no significant differences among treatments in initial or final weights, there was an effect of treatment on the change of weights (*H* = 13.46, *df* = 4,68, *p* < 0.01). In comparison to fish fed twice a day, fish fed every two days gained less weight (*p* < 0.01). Sample sizes vary slightly among variables due to technical problems with the behavior analysis software and one mortality. Please refer to the supplementary information for additional statistical details.

## Discussion

Here we show that the feeding regimen may affect zebrafish behavior. Fish fed 3 h before behavioral evaluation presented a different behavioral pattern in the NTT. Similarly, different feeding frequencies also change behavior.

In Experiment 1, fish fed 3 h before behavioral testing were less anxious and demonstrated hypoactivity, as revealed by the shorter distance travelled compared to other treatments (Fig. 2A), more time spent in the top compared to fish fed 24 h before testing (Fig. 2D), and shorter latency to enter this zone (Fig. 2E) compared to fish fed 24 h and 48 h before NTT. The marked hypoactivity in fish fed 3 h before behavioral testing could be attributed to a post-prandial alkaline tide, a metabolic alkalosis created by gastric hydrochloric acid secretion during digestion. This well-known phenomenon in mammalian species has been verified in freshwater fish such as rainbow trout (*Oncorhynchus mykiss*) (Bucking & Wood, 2008; Bucking et al., 2009; Bucking, Landman & Wood, 2010). Thus, the alkaline tide may be a mechanism underlying the different outcomes in the NTT test. Another possible mechanism is a postprandial sparing of intestinal blood flow to support digestion, thereby limiting the allocation of blood flow to locomotory muscles as postulated by Thorarensen & Farrell (2006) in exercised chinook salmon (*Oncorhynchus tshawytscha*). Similarly, increased metabolic rate during digestion has also been correlated with altered swimming performance in several cyprinidae species (Nie & Fu, 2017; Nie, Cao & Fu, 2017).

In Experiment 2, fish fed once a day presented anxiety-like behavior compared to those that received feeding twice a day. This was evident from the short time spent at the tank top, higher latency to enter the top zone, and longer time spent at the tank bottom. However, it is unclear why fish fed every two days did not demonstrate this pattern. The response may be related to metabolism. Studies have shown that plasma levels of glucose are correlated with psychological and behavioral alterations (Al Hayek et al., 2015; Gold, 2015), diet interventions, and anxiety levels (Agarwal et al., 2015). In addition, caloric restriction is associated with changes in behavior (Mansur, Brietzke & McIntyre, 2015) and stress responsiveness (Tomiyama et al., 2010; Pankevich et al., 2010). However, in the present study, all fish received the same amount of calories, and only the frequency varied. Thus, we hypothesize that the abrupt fall in glucose and glycogen levels (Fig. 4A) is the main factor responsible for the behavioral changes in fish fed once a day but not in fish fed every two days, compared to fish fed twice a day. However, we cannot exclude the possibility that fish in the different treatments consumed different amounts of food. A lower weight gain in fish fed every two days in comparison to fish fed twice a day may indicate that they were unable to consume the relatively large amount of food during the available time.

Metabolically, fish fed once a day presented altered levels of glucose, glycogen, and lactate; denoting a clear effect of feeding regimen on intermediary carbohydrate metabolism. Zebrafish fed once a day demonstrated a decreased level of whole body glucose compared to those fed twice a day. Interestingly, zebrafish fed at 48 h frequency presented glucose levels similar to those fed twice a day. A possible explanation is based on the stressful effect of fasting, which may potentiate the glucocorticoid effects of cortisol (Groff & Zinkl, 1999; Barcellos et al., 2010). Data about glucose dynamics in zebrafish are scarce, but a similar pattern was found in previous research (Barcellos et al., 2010).

Similarly, fish fed once a day demonstrated reduced glycogen levels, probably due to the cortisol effect mentioned above (Barcellos et al., 2010; Eames et al., 2010). Fasting periods tend to activate the adaptive utilization of alternative energy sources (gluconeogenesis via proteolysis) in the organism (Craig & Moon, 2011; Marqueze et al., 2017). In this regard, the normal glycogen level measured in fish fed at 48 h frequency reinforced adaptation of the organism to frequent fasting (Barcellos et al., 2010). The increased levels of lactate measured in fish fed once a day and at 48 h frequency could be attributed to anaerobic glycolysis caused by fasting (Campbell & Farrell, 2014).

Mechanistically, we suggest that the metabolism changes caused by the feeding regimen are causative factors of the behavioral changes observed. Since the activity level and exploratory behavior are associated with energy expenditure (Speakman & Selman, 2003), the metabolic changes caused by the feeding regimen may be associated with the changes observed in fish performance on the NTT. In this regard, exploratory activity, as well as responsiveness to novelty and stress, are highly associated with animal personality and energy metabolism (Barreto & Volpato, 2011; Careau et al., 2018).

We cannot exclude the possibility that the feeding regimen, metabolism alterations, and behavioral changes observed in the present study might not be causally linked. In rodents, food restriction can produce changes in stress responsiveness that alters metabolism and nervous system activity. These factors can produce behavioral changes and other adjustments (Pankevich et al., 2010). The link between acute stress and food intake behavior has been studied in fish and may occur via ghrelin signaling (Pankhurst, King & Ludke, 2008; Upton & Riley, 2013). Since the aquarium set-up used in Experiment 2 (Fig. 1) may have been stressful (Débora Fior, personal communication), acute stress may have been a co-causative factor of the metabolic changes observed. Furthermore, physical isolation in Experiment 2 may have interfered with the behavior of fish. Indeed, isolation causes changes in the stress response (Giacomini et al., 2015), and glucocorticoids released in the stress response can interfere with intermediary metabolism. However, this hypothesis seems less likely as the apparatus allowed the chemical and visual contact between fish.

In Experiment 1, fish were maintained in groups of three, since isolation is a stressful condition for zebrafish (Giacomini et al., 2015) and may cause changes in behavioral performance (Pagnussat et al., 2013). On the other hand, fish grouped for a 10-day period can establish shoaling (Spence et al., 2008; Buske & Gerlai, 2011) and hierarchical organization (Paull et al., 2010) that can modulate aggression and promote competition for food (Grant & Kramer, 1992), which interferes with the amount of food consumed by each fish. However, we observed fish during the feeding period and observed that all fish ate food in similar amounts.

Regardless of the mechanism that induces the behavioral changes, the results of the present study may have practical applications since the high variability of the results between different laboratories may be related to the different feeding protocols employed. On the other hand, since some behavioral parameters were not altered, variation in feeding regime is possible without behavioral effects. Thus, if issues regarding the interval between the last feeding and tests are not considered, the differences (or its absence) in the results may be erroneously interpreted and related to treatments or conditions, whereas the differences could be due to artifacts from the experimental protocol. Although we did not compare data of Experiment 1 and Experiment 2, fish from the second experiment seems to be more anxious reinforcing our hypothesis that differences in feeding protocols can cause discrepancies in behavioral outcomes.

## Conclusion

Fish fed 3 h before behavioral testing demonstrated anxiolytic behavior with marked hypoactivity and longer time spent in the top zone of the tank. In addition, fish fed once a day demonstrated anxiety-like behavior compared to those fed twice a day. Based on these data, we conclude that feeding regimen modulates fish behavior. This issue may be considered as a source of variation in the results of laboratories around the world. More studies are needed to replicate these findings and confirm whether different feeding regimens can influence behavioral, biochemical and molecular parameters in zebrafish.

##  Supplemental Information

10.7717/peerj.5343/supp-1 Supplemental Information 1 Data and statistics

## Additional Information and Declarations

### Data Availability

The following information was supplied regarding data availability: The data are included in Supplemental Information 1.

## References

[ref-1] Abreu MS, Giacomini ACV, Gusso D, Rosa JGS, Koakoski G, Kalichak F, Idalêncio R, Oliveira TA, Barcellos HHA, Bonan CD, Barcellos LJG (2016). Acute exposure to waterborne psychoactive drugs attract zebrafish. Comparative Biochemistry and Physiology. Toxicology & Pharmacology.

[ref-2] Abreu MS, Oliveira TA, Koakoski G, Barreto RE, Barcellos LJG (2018). Modulation of cortisol responses to an acute stressor in zebrafish visually exposed to heterospecific fish during development. Zebrafish.

[ref-3] Agarwal U, Mishra S, Xu J, Levin S, Gonzales J, Barnard ND (2015). A multicenter randomized controlled trial of a nutrition intervention program in a multiethnic adult population in the corporate setting reduces depression and anxiety and improves quality of life: the GEICO study. American Journal of Health Promotion.

[ref-4] Al Hayek AA, Robert AA, Braham RB, Issa BA, Al Sabaan FS (2015). Predictive risk factors for fear of hypoglycemia and anxiety-related emotional disorders among adolescents with Type 1 diabetes. Medical Principles and Practice.

[ref-5] Barbazuk WB, Korf I, Kadavi C, Heyen J, Tate S, Wun E, Bedell JA, McPherson JD, Johnson SL (2000). The syntenic relationship of the zebrafish and human genomes. Genome Research.

[ref-6] Barcellos HH de A, Kalichak F, Da Rosa JGS, Oliveira TA, Koakoski G, Idalencio R, De Abreu MS, Giacomini ACV, Fagundes M, Variani C, Rossini M, Piato AL, Barcellos LJG (2016). Waterborne aripiprazole blunts the stress response in zebrafish. Scientific Reports.

[ref-7] Barcellos LJG, Marqueze A, Trapp M, Quevedo RM, Ferreira D (2010). The effects of fasting on cortisol, blood glucose and liver and muscle glycogen in adult jundiá *Rhamdia quelen*. Aquaculture.

[ref-8] Barreto RE, Volpato GL (2011). Ventilation rates indicate stress-coping styles in Nile tilapia. Journal of Biosciences.

[ref-9] Boitard C, Etchamendy N, Sauvant J, Aubert A, Tronel S, Marighetto A, Layé S, Ferreira G (2012). Juvenile, but not adult exposure to high-fat diet impairs relational memory and hippocampal neurogenesis in mice. Hippocampus.

[ref-10] Bucking C, Fitzpatrick JL, Nadella SR, Wood CM (2009). Post-prandial metabolic alkalosis in the seawater-acclimated trout: the alkaline tide comes in. The Journal of Experimental Biology.

[ref-11] Bucking C, Landman MJ, Wood CM (2010). The role of the kidney in compensating the alkaline tide, electrolyte load, and fluid balance disturbance associated with feeding in the freshwater rainbow trout, *Oncorhynchus mykiss*. Comparative Biochemistry and Physiology Part A: Molecular & Integrative Physiology.

[ref-12] Bucking C, Wood CM (2008). The alkaline tide and ammonia excretion after voluntary feeding in freshwater rainbow trout. The Journal of Experimental Biology.

[ref-13] Buske C, Gerlai R (2011). Shoaling develops with age in Zebrafish (*Danio rerio*). Progress in Neuro-Psychopharmacology & Biological Psychiatry.

[ref-14] Campbell MK, Farrell SO (2014). Biochemistry.

[ref-15] Canzian J, Fontana BD, Quadros VA, Rosemberg DB (2017). Conspecific alarm substance differently alters group behavior of zebrafish populations: putative involvement of cholinergic and purinergic signaling in anxiety- and fear-like responses. Behavioural Brain Research.

[ref-16] Careau V, Thomas D, Humphries MM, Réale D (2018). Energy metabolism and animal personality. Oikos.

[ref-17] Craig PM, Moon TW (2011). Fasted zebrafish mimic genetic and physiological responses in mammals: a model for obesity and diabetes?. Zebrafish.

[ref-18] Da Rosa JGS, Barcellos HH de A, Idalencio R, Marqueze A, Fagundes M, Rossini M, Variani C, Balbinoti F, Tietböhl TMH, Rosemberg DB, Barcellos LJG (2017). Just keep swimming: neuroendocrine, metabolic, and behavioral changes after a forced swimming test in zebrafish. Zebrafish.

[ref-19] Dong GF, Yang YO, Yao F, Chen L, Bu FY, Li PC, Huang F, Yu DH (2015). Individual variations and interrelationships in feeding rate, growth rate, and spontaneous activity in hybrid tilapia (Oreochromis niloticus × O. aureus) at different feeding frequencies. Journal of Applied Ichthyology.

[ref-20] Dwyer KS, Brown JA, Parrish C, Lall SP (2002). Feeding frequency affects food consumption, feeding pattern and growth of juvenile yellowtail flounder (*Limanda ferruginea*). Aquaculture.

[ref-21] Eames SC, Philipson LH, Prince VE, Kinkel MD (2010). Blood sugar measurement in zebrafish reveals dynamics of glucose homeostasis. Zebrafish.

[ref-22] Egan RJ, Bergner CL, Hart PC, Cachat JM, Canavello PR, Elegante MF, Elkhayat SI, Bartels BK, Tien AK, Tien DH, Mohnot S, Beeson E, Glasgow E, Amri H, Zukowska Z, Kalueff AV (2009). Understanding behavioral and physiological phenotypes of stress and anxiety in zebrafish. Behavioural Brain Research.

[ref-23] Forsatkar MN, Nematollahi MA, Rafiee G, Farahmand H, Lawrence C (2017). Effects of the prebiotic mannan-oligosaccharide on the stress response of feed deprived zebrafish (*Danio rerio*). Physiology & Behavior.

[ref-24] Frenzl B, Stien LH, Cockerill D, Oppedal F, Richards RH, Shinn AP, Bron JE, Migaud H (2014). Manipulation of farmed Atlantic salmon swimming behaviour through the adjustment of lighting and feeding regimes as a tool for salmon lice control. Aquaculture.

[ref-25] Giacomini ACVV, De Abreu MS, Koakoski G, Idalêncio R, Kalichak F, Oliveira TA, Da Rosa JGS, Gusso D, Piato AL, Barcellos LJG (2015). My stress, our stress: blunted cortisol response to stress in isolated housed zebrafish. Physiology & Behavior.

[ref-26] Gold PW (2015). The organization of the stress system and its dysregulation in depressive illness. Molecular Psychiatry.

[ref-27] Grant JWA, Kramer DL (1992). Temporal clumping of food arrival reduces its monopolization and defence by zebrafish, *Brachydanio rerio*. Animal Behaviour.

[ref-28] Groff JM, Zinkl JG (1999). Hematology and clinical chemistry of cyprinid fish. Common carp and goldfish. The Veterinary Clinics of North America. Exotic Animal Practice.

[ref-29] Jones HAC, Noble C, Damsgård B, Pearce GP (2018). Evaluating the effects of a short-term feed restriction period on the behavior and welfare of Atlantic Salmon, *Salmo salar*, parr using social network analysis and fin damage. Journal of the World Aquaculture Society.

[ref-30] Kalichak F, Idalencio R, Rosa JGS, De Oliveira TA, Koakoski G, Gusso D, De Abreu MS, Giacomini ACV, Barcellos HHA, Fagundes M, Piato AL, Barcellos LJG (2016). Waterborne psychoactive drugs impair the initial development of Zebrafish. Environmental Toxicology and Pharmacology.

[ref-31] Kalueff AV, Gebhardt M, Stewart AM, Cachat JM, Brimmer M, Chawla JS, Craddock C, Kyzar EJ, Roth A, Landsman S, Gaikwad S, Robinson K, Baatrup E, Tierney K, Shamchuk A, Norton W, Miller N, Nicolson T, Braubach O, Gilman CP, Pittman J, Rosemberg DB, Gerlai R, Echevarria D, Lamb E, Neuhauss SCF, Weng W, Bally-Cuif L, Schneider H (2013). Towards a comprehensive catalog of zebrafish behavior 1.0 and beyond. Zebrafish.

[ref-32] Kirsten K, Fior D, Kreutz LC, Barcellos LJG (2018). First description of behavior and immune system relationship in fish. Scientific Reports.

[ref-33] Kysil EV, Meshalkina DA, Frick EE, Echevarria DJ, Rosemberg DB, Maximino C, Lima MG, Abreu MS, Giacomini AC, Barcellos LJG, Song C, Kalueff AV (2017). Comparative analyses of zebrafish anxiety-like behavior using conflict-based novelty tests. Zebrafish.

[ref-34] Lawrence C (2007). The husbandry of zebrafish (*Danio rerio*): a review. Aquaculture.

[ref-35] Lee S-M, Hwang U-G, Cho SH (2000). Effects of feeding frequency and dietary moisture content on growth, body composition and gastric evacuation of juvenile Korean rockfish (*Sebastes schlegeli*). Aquaculture.

[ref-36] Mansur RB, Brietzke E, McIntyre RS (2015). Is there a “metabolic-mood syndrome”? A review of the relationship between obesity and mood disorders. Neuroscience and Biobehavioral Reviews.

[ref-37] Marqueze A, Garbino CF, Trapp M, Kucharski LC, Fagundes M, Ferreira D, Koakoski G, Rosa JGS (2017). Protein and lipid metabolism adjustments in silver catfish (*Rhamdia quelen*) during different periods of fasting and refeeding. Brazilian Journal of Biology.

[ref-38] McDougall M, Choi J, Magnusson K, Truong L, Tanguay R, Traber MG (2017). Chronic vitamin E deficiency impairs cognitive function in adult zebrafish via dysregulation of brain lipids and energy metabolism. Free Radical Biology & Medicine.

[ref-39] Mocelin R, Herrmann AP, Marcon M, Rambo CL, Rohden A, Bevilaqua F, De Abreu MS, Zanatta L, Elisabetsky E, Barcellos LJG, Lara DR, Piato AL (2015). N-acetylcysteine prevents stress-induced anxiety behavior in zebrafish. Pharmacology, Biochemistry, and Behavior.

[ref-40] Nie LJ, Cao ZD, Fu SJ (2017). Digesting or swimming? Integration of the postprandial metabolism, behavior and locomotion in a frequently foraging fish. Comparative Biochemistry and Physiology Part A: Molecular & Integrative Physiology.

[ref-41] Nie LJ, Fu SJ (2017). Metabolic, behavioral, and locomotive effects of feeding in five cyprinids with different habitat preferences. Fish Physiology and Biochemistry.

[ref-42] Norton W, Bally-Cuif L (2010). Adult zebrafish as a model organism for behavioural genetics. BMC Neuroscience.

[ref-43] O’Brine TM, Vrtělová J, Snellgrove DL, Davies SJ, Sloman KA (2015). Growth, oxygen consumption, and behavioral responses of *Danio rerio* to variation in dietary protein and lipid levels. Zebrafish.

[ref-44] Pagnussat N, Piato AL, Schaefer IC, Blank M, Tamborski AR, Guerim LD, Bonan CD, Vianna MRM, Lara DR (2013). One for all and all for one: the importance of shoaling on behavioral and stress responses in zebrafish. Zebrafish.

[ref-45] Pankevich DE, Teegarden SL, Hedin AD, Jensen CL, Bale TL (2010). Caloric restriction experience reprograms stress and orexigenic pathways and promotes binge eating. The Journal of Neuroscience.

[ref-46] Pankhurst NW, King HR, Ludke SL (2008). Relationship between stress, feeding and plasma ghrelin levels in rainbow trout, *Oncorhynchus mykiss*. Marine and Freshwater Behaviour and Physiology.

[ref-47] Paull GC, Filby AL, Giddins HG, Coe TS, Hamilton PB, Tyler CR (2010). Dominance hierarchies in zebrafish (*Danio rerio*) and their relationship with reproductive success. Zebrafish.

[ref-48] Piato ÂL, Capiotti KM, Tamborski AR, Oses JP, Barcellos LJG, Bogo MR, Lara DR, Vianna MR, Bonan CD (2011). Unpredictable chronic stress model in zebrafish (*Danio rerio*): behavioral and physiological responses. Progress in Neuro-Psychopharmacology & Biological Psychiatry.

[ref-49] Rambo CL, Mocelin R, Marcon M, Villanova D, Koakoski G, De Abreu MS, Oliveira TA, Barcellos LJG, Piato AL, Bonan CD (2016). Gender differences in aggression and cortisol levels in zebrafish subjected to unpredictable chronic stress. Physiology & Behavior.

[ref-50] Réale D, Reader SM, Sol D, McDougall PT, Dingemanse NJ (2007). Integrating animal temperament within ecology and evolution. Biological Reviews of the Cambridge Philosophical Society.

[ref-51] Reolon GK, De Melo GM, Da Rosa JGDS, Barcellos LJG, Bonan CD (2018). Sex and the housing: effects on behavior, cortisol levels and weight in zebrafish. Behavioural Brain Research.

[ref-52] Rosa LV, Ardais AP, Costa FV, Fontana BD, Quadros VA, Porciúncula LO, Rosemberg DB (2018). Different effects of caffeine on behavioral neurophenotypes of two zebrafish populations. Pharmacology, Biochemistry, and Behavior.

[ref-53] Santos da Rosa JG, Alcântara Barcellos HH de, Fagundes M, Variani C, Rossini M, Kalichak F, Koakoski G, Acosta Oliveira T, Idalencio R, Frandoloso R, Piato AL, José Gil Barcellos L (2017). Muscarinic receptors mediate the endocrine-disrupting effects of an organophosphorus insecticide in zebrafish. Environmental Toxicology.

[ref-54] Shi C, Liu Y, Yi M, Zheng J, Tian H, Du Y, Li X, Sun G (2017). Comparison of time-restricted and ad libitum self-feeding on the growth, feeding behavior and daily digestive enzyme profiles of Atlantic salmon. Chinese Journal of Oceanology and Limnology.

[ref-55] Speakman JR, Selman C (2003). Physical activity and resting metabolic rate. The Proceedings of the Nutrition Society.

[ref-56] Spence R, Gerlach G, Lawrence C, Smith C (2008). The behaviour and ecology of the zebrafish, *Danio rerio*. Biological Reviews of the Cambridge Philosophical Society.

[ref-57] Stewart A, Wong K, Cachat J, Gaikwad S, Kyzar E, Wu N, Hart P, Piet V, Utterback E, Elegante M, Tien D, Kalueff AV (2011). Zebrafish models to study drug abuse-related phenotypes. Reviews in the Neurosciences.

[ref-58] Thorarensen H, Farrell A (2006). Postprandial Intestinal Blood Flow, Metabolic Rates, and Exercise in Chinook Salmon (Oncorhynchus tshawytscha). Physiological and Biochemical Zoology.

[ref-59] Tierney KB (2011). Behavioural assessments of neurotoxic effects and neurodegeneration in zebrafish. Biochimica Et Biophysica Acta.

[ref-60] Tomiyama AJ, Mann T, Vinas D, Hunger JM, Dejager J, Taylor SE (2010). Low calorie dieting increases cortisol. Psychosomatic Medicine.

[ref-61] Upton KR, Riley LG (2013). Acute stress inhibits food intake and alters ghrelin signaling in the brain of tilapia (*Oreochromis mossambicus*). Domestic Animal Endocrinology.

[ref-62] Van Handel E (1965). Estimation of glycogen in small amounts of tissue. Analytical Biochemistry.

[ref-63] Wilson D, Clark AB, Coleman K, Dearstyne T (1994). Shyness and boldness in humans and other animals. Trends in Ecology & Evolution.

