# Supplementary material for: Feeding regimen modulates zebrafish behavior
Source: PeerJ. 2018 Aug 3;6:e5343. doi: 10.7717/peerj.5343 (PMC6080598; doi:10.7717/peerj.5343)
Supplement: Supplemental Information 1 [file peerj-06-5343-s001.docx]

**Dametto et al.**

**Feeding regime modulates zebrafish behavior**

**Data and statistics**

**FIGURE 2**

Data and statistics of figure 2 (A) – total distance travelled (m). Each data is the mean of three fish.

| Aquarium number | 30min | 3h | 6h | 12h | 24h | 48h |
| --- | --- | --- | --- | --- | --- | --- |
| 1 | 15.87 | 7.29 | 18.24 | 20.08 | 15.52 | 20.11 |
| 2 | 19.50 | 3.16 | 15.26 | 21.28 | 15.68 | 14.57 |
| 3 | 13.44 | 6.15 | 18.57 | 22.38 | 19.24 | 18.75 |
| 4 | 22.12 | 6.66 | 15.65 | 15.51 | 14.77 | 14.68 |

| Table Analyzed | | | distance | | |  |  |  |  |
| --- | --- | --- | --- | --- | --- | --- | --- | --- | --- |
|  | | |  | | |  |  |  |  |
| ANOVA summary | | |  | | |  |  |  |  |
| F | | | 13,93 | | |  |  |  |  |
| P value | | | < 0,0001 | | |  |  |  |  |
| P value summary | | | **** | | |  |  |  |  |
| Are differences among means statistically significant? (P < 0.05) | | | Yes | | |  |  |  |  |
| R square | | | 0,7946 | | |  |  |  |  |
|  | | |  | | |  |  |  |  |
| Brown-Forsythe test | | |  | | |  |  |  |  |
| F (DFn, DFd) | | | 1,154 (5, 18) | | |  |  |  |  |
| P value | | | 0,3689 | | |  |  |  |  |
| P value summary | | | ns | | |  |  |  |  |
| Significantly different standard deviations? (P < 0.05) | | | No | | |  |  |  |  |
|  | | |  | | |  |  |  |  |
| Bartlett's test | | |  | | |  |  |  |  |
| Bartlett's statistic (corrected) | | | 2,772 | | |  |  |  |  |
| P value | | | 0,7352 | | |  |  |  |  |
| P value summary | | | ns | | |  |  |  |  |
| Significantly different standard deviations? (P < 0.05) | | | No | | |  |  |  |  |
|  | | |  | | |  |  |  |  |
| ANOVA table | | | SS | | | DF | MS | F (DFn, DFd) | P value |
| Treatment (between columns) | | | 489,4 | | | 5 | 97,87 | F (5, 18) = 13,93 | P < 0,0001 |
| Residual (within columns) | | | 126,5 | | | 18 | 7,027 |  |  |
| Total | | | 615,8 | | | 23 |  |  |  |
|  | | |  | | |  |  |  |  |
|  | | |  | | |  |  |  |  |
|  | | |  | | |  |  |  |  |
| Number of families | 1 |  | |  |  |  |  |  |  |
| Number of comparisons per family | 15 |  | |  |  |  |  |  |  |
| Alpha | 0,05 |  | |  |  |  |  |  |  |
|  |  |  | |  |  |  |  |  |  |
| Holm-Sidak's multiple comparisons test | Mean Diff, | Significant? | | Summary |  |  |  |  |  |
|  |  |  | |  |  |  |  |  |  |
| 30min vs. 3h | 11,92 | Yes | | **** |  |  |  |  |  |
| 30min vs. 6h | 0,8025 | No | | ns |  |  |  |  |  |
| 30min vs. 12h | -2,080 | No | | ns |  |  |  |  |  |
| 30min vs. 24h | 1,430 | No | | ns |  |  |  |  |  |
| 30min vs. 48h | 0,7050 | No | | ns |  |  |  |  |  |
| 3h vs. 6h | -11,12 | Yes | | *** |  |  |  |  |  |
| 3h vs. 12h | -14,00 | Yes | | **** |  |  |  |  |  |
| 3h vs. 24h | -10,49 | Yes | | *** |  |  |  |  |  |
| 3h vs. 48h | -11,21 | Yes | | *** |  |  |  |  |  |
| 6h vs. 12h | -2,883 | No | | ns |  |  |  |  |  |
| 6h vs. 24h | 0,6275 | No | | ns |  |  |  |  |  |
| 6h vs. 48h | -0,09750 | No | | ns |  |  |  |  |  |
| 12h vs. 24h | 3,510 | No | | ns |  |  |  |  |  |
| 12h vs. 48h | 2,785 | No | | ns |  |  |  |  |  |
| 24h vs. 48h | -0,7250 | No | | ns |  |  |  |  |  |

Data and statistics of figure 2 (B) – crossing number (Number of crossing per 6 minutes). Each data is the mean of three fish.

| Aquarium number | 30min | 3h | 6h | 12h | 24h | 48h |
| --- | --- | --- | --- | --- | --- | --- |
| 1 | 127 | 61 | 117 | 156 | 142 | 58 |
| 2 | 172 | 22 | 133 | 217 | 85 | 123 |
| 3 | 110 | 16 | 109 | 145 | 125 | 102 |
| 4 | 175 | 47 | 118 | 43 | 94 | 81 |

| Table Analyzed | crossings |  |  |  |  |
| --- | --- | --- | --- | --- | --- |
|  |  |  |  |  |  |
| ANOVA summary |  |  |  |  |  |
| F | 4,641 |  |  |  |  |
| P value | 0,0068 |  |  |  |  |
| P value summary | ** |  |  |  |  |
| Are differences among means statistically significant? (P < 0.05) | Yes |  |  |  |  |
| R square | 0,5632 |  |  |  |  |
|  |  |  |  |  |  |
| Brown-Forsythe test |  |  |  |  |  |
| F (DFn, DFd) | 1,453 (5, 18) |  |  |  |  |
| P value | 0,2537 |  |  |  |  |
| P value summary | ns |  |  |  |  |
| Significantly different standard deviations? (P < 0.05) | No |  |  |  |  |
|  |  |  |  |  |  |
| Bartlett's test |  |  |  |  |  |
| Bartlett's statistic (corrected) | 10,47 |  |  |  |  |
| P value | 0,0630 |  |  |  |  |
| P value summary | ns |  |  |  |  |
| Significantly different standard deviations? (P < 0.05) | No |  |  |  |  |
|  |  |  |  |  |  |
| ANOVA table | SS | DF | MS | F (DFn, DFd) | P value |
| Treatment (between columns) | 32088 | 5 | 6418 | F (5, 18) = 4,641 | P = 0,0068 |
| Residual (within columns) | 24890 | 18 | 1383 |  |  |
| Total | 56978 | 23 |  |  |  |

| Number of families | 1 |  |  |
| --- | --- | --- | --- |
| Number of comparisons per family | 15 |  |  |
| Alpha | 0,05 |  |  |
|  |  |  |  |
| Holm-Sidak's multiple comparisons test | Mean Diff, | Significant? | Summary |
|  |  |  |  |
| 30min vs. 3h | 109,5 | Yes | ** |
| 30min vs. 6h | 26,75 | No | ns |
| 30min vs. 12h | 5,750 | No | ns |
| 30min vs. 24h | 34,50 | No | ns |
| 30min vs. 48h | 55,00 | No | ns |
| 3h vs. 6h | -82,75 | No | ns |
| 3h vs. 12h | -103,8 | Yes | * |
| 3h vs. 24h | -75,00 | No | ns |
| 3h vs. 48h | -54,50 | No | ns |
| 6h vs. 12h | -21,00 | No | ns |
| 6h vs. 24h | 7,750 | No | ns |
| 6h vs. 48h | 28,25 | No | ns |
| 12h vs. 24h | 28,75 | No | ns |
| 12h vs. 48h | 49,25 | No | ns |
| 24h vs. 48h | 20,50 | No | ns |

Data and statistics of figure 2 (C) – rotations (number of rotations per 6 minutes). Each data is the mean of three fish.

| Aquarium number | 30min | 3h | 6h | 12h | 24h | 48h |
| --- | --- | --- | --- | --- | --- | --- |
| 1 | 26 | 6 | 23 | 17 | 22 | 15 |
| 2 | 21 | 5 | 12 | 30 | 28 | 17 |
| 3 | 18 | 4 | 21 | 28 | 24 | 19 |
| 4 | 29 | 10 | 23 | 14 | 27 | 16 |

| Table Analyzed | rotations |
| --- | --- |
|  |  |
| Kruskal-Wallis test |  |
| P value | 0,0162 |
| Exact or approximate P value? | Approximate |
| P value summary | * |
| Do the medians vary signif. (P < 0.05) | Yes |
| Number of groups | 6 |
| Kruskal-Wallis statistic | 13,91 |
|  |  |
| Data summary |  |
| Number of treatments (columns) | 6 |
| Number of values (total) | 24 |

| Number of families | 1 |  |  |
| --- | --- | --- | --- |
| Number of comparisons per family | 15 |  |  |
| Alpha | 0,05 |  |  |
|  |  |  |  |
| Dunn's multiple comparisons test | Mean rank diff, | Significant? | Summary |
|  |  |  |  |
| 30min vs. 3h | 14,13 | No | ns |
| 30min vs. 6h | 3,750 | No | ns |
| 30min vs. 12h | 1,375 | No | ns |
| 30min vs. 24h | -2,000 | No | ns |
| 30min vs. 48h | 7,500 | No | ns |
| 3h vs. 6h | -10,38 | No | ns |
| 3h vs. 12h | -12,75 | No | ns |
| 3h vs. 24h | -16,13 | Yes | ** |
| 3h vs. 48h | -6,625 | No | ns |
| 6h vs. 12h | -2,375 | No | ns |
| 6h vs. 24h | -5,750 | No | ns |
| 6h vs. 48h | 3,750 | No | ns |
| 12h vs. 24h | -3,375 | No | ns |
| 12h vs. 48h | 6,125 | No | ns |
| 24h vs. 48h | 9,500 | No | ns |

Data and statistics of figure 2 (D) – time spent at the top zone (s). Each data is the mean of three fish.

| Aquarium number | 30min | 3h | 6h | 12h | 24h | 48h |
| --- | --- | --- | --- | --- | --- | --- |
| 1 | 62.17 | 274.70 | 54.37 | 31.27 | 65.20 | 18.53 |
| 2 | 87.20 | 90.37 | 76.83 | 95.30 | 41.83 | 29.23 |
| 3 | 69.50 | 179.43 | 100.70 | 47.43 | 21.10 | 59.66 |
| 4 | 50.43 | 98.25 | 49.67 | 12.75 | 18.14 | 43.07 |

| Table Analyzed | time top |
| --- | --- |
|  |  |
| Kruskal-Wallis test |  |
| P value | 0,0194 |
| Exact or approximate P value? | Approximate |
| P value summary | * |
| Do the medians vary signif. (P < 0.05) | Yes |
| Number of groups | 6 |
| Kruskal-Wallis statistic | 13,46 |
|  |  |
| Data summary |  |
| Number of treatments (columns) | 6 |
| Number of values (total) | 24 |

| Number of families | 1 |  |  |
| --- | --- | --- | --- |
| Number of comparisons per family | 15 |  |  |
| Alpha | 0,05 |  |  |
|  |  |  |  |
| Dunn's multiple comparisons test | Mean rank diff, | Significant? | Summary |
|  |  |  |  |
| 30min vs. 3h | -7,000 | No | ns |
| 30min vs. 6h | -0,5000 | No | ns |
| 30min vs. 12h | 5,750 | No | ns |
| 30min vs. 24h | 7,750 | No | ns |
| 30min vs. 48h | 7,500 | No | ns |
| 3h vs. 6h | 6,500 | No | ns |
| 3h vs. 12h | 12,75 | No | ns |
| 3h vs. 24h | 14,75 | Yes | ** |
| 3h vs. 48h | 14,50 | No | ns |
| 6h vs. 12h | 6,250 | No | ns |
| 6h vs. 24h | 8,250 | No | ns |
| 6h vs. 48h | 8,000 | No | ns |
| 12h vs. 24h | 2,000 | No | ns |
| 12h vs. 48h | 1,750 | No | ns |
| 24h vs. 48h | -0,2500 | No | ns |

Data and statistics of figure 2 (E) – latency to the first entry in the top zone (s). Each data is the mean of three fish.

| Aquarium number | 30min | 3h | 6h | 12h | 24h | 48h |
| --- | --- | --- | --- | --- | --- | --- |
| 1 | 36 | 12 | 72 | 118 | 69 | 120 |
| 2 | 11 | 20 | 35 | 40 | 128 | 104 |
| 3 | 97 | 18 | 66 | 59 | 151 | 62 |
| 4 | 70 | 66 | 75 | 129 | 91 | 128 |

| Table Analyzed | latency to top |  |  |  |  |
| --- | --- | --- | --- | --- | --- |
|  |  |  |  |  |  |
| ANOVA summary |  |  |  |  |  |
| F | 3,612 |  |  |  |  |
| P value | 0,0194 |  |  |  |  |
| P value summary | * |  |  |  |  |
| Are differences among means statistically significant? (P < 0.05) | Yes |  |  |  |  |
| R square | 0,5008 |  |  |  |  |
|  |  |  |  |  |  |
| Brown-Forsythe test |  |  |  |  |  |
| F (DFn, DFd) | 1,518 (5, 18) |  |  |  |  |
| P value | 0,2335 |  |  |  |  |
| P value summary | ns |  |  |  |  |
| Significantly different standard deviations? (P < 0.05) | No |  |  |  |  |
|  |  |  |  |  |  |
| Bartlett's test |  |  |  |  |  |
| Bartlett's statistic (corrected) | 2,370 |  |  |  |  |
| P value | 0,7960 |  |  |  |  |
| P value summary | ns |  |  |  |  |
| Significantly different standard deviations? (P < 0.05) | No |  |  |  |  |
|  |  |  |  |  |  |
| ANOVA table | SS | DF | MS | F (DFn, DFd) | P value |
| Treatment (between columns) | 19575 | 5 | 3915 | F (5, 18) = 3,612 | P = 0,0194 |
| Residual (within columns) | 19510 | 18 | 1084 |  |  |
| Total | 39085 | 23 |  |  |  |

| Number of families | 1 |  |  |  |
| --- | --- | --- | --- | --- |
| Number of comparisons per family | 15 |  |  |  |
| Alpha | 0,05 |  |  |  |
|  |  |  |  |  |
| Tukey's multiple comparisons test | Mean Diff, | 95% CI of diff, | Significant? | Summary |
|  |  |  |  |  |
| 30min vs. 3h | 24,50 | -49,48 to 98,48 | No | ns |
| 30min vs. 6h | -8,500 | -82,48 to 65,48 | No | ns |
| 30min vs. 12h | -33,00 | -107,0 to 40,98 | No | ns |
| 30min vs. 24h | -56,25 | -130,2 to 17,73 | No | ns |
| 30min vs. 48h | -50,00 | -124,0 to 23,98 | No | ns |
| 3h vs. 6h | -33,00 | -107,0 to 40,98 | No | ns |
| 3h vs. 12h | -57,50 | -131,5 to 16,48 | No | ns |
| 3h vs. 24h | -80,75 | -154,7 to -6,767 | Yes | * |
| 3h vs. 48h | -74,50 | -148,5 to -0,5168 | Yes | * |
| 6h vs. 12h | -24,50 | -98,48 to 49,48 | No | ns |
| 6h vs. 24h | -47,75 | -121,7 to 26,23 | No | ns |
| 6h vs. 48h | -41,50 | -115,5 to 32,48 | No | ns |
| 12h vs. 24h | -23,25 | -97,23 to 50,73 | No | ns |
| 12h vs. 48h | -17,00 | -90,98 to 56,98 | No | ns |
| 24h vs. 48h | 6,250 | -67,73 to 80,23 | No | ns |

**FIGURE 3**

Data and statistics of figure 3 (A) – total distance travelled (m)

| 6X | 4X | 2X | 1X | 1X/2D |
| --- | --- | --- | --- | --- |
| 14.442 | 14.752 | 19.037 | 23.350 | 5.842 |
| 10.883 | 15.190 | 13.705 | 15.788 | 17.554 |
| 19.607 | 19.137 | 20.964 | 17.976 | 13.989 |
| 25.001 | 17.315 | 27.764 | 11.090 | 8.632 |
| 10.524 | 19.440 | 14.078 | 14.251 | 17.053 |
| 14.351 | 19.928 | 26.624 | 26.830 | 23.202 |
| 25.361 | 16.560 | 17.335 | 19.076 | 26.132 |
| 13.074 | 13.586 | 19.780 | 16.104 | 10.140 |
| 23.710 | 16.397 | 13.508 | 15.380 | 13.968 |
| 6.475 | 21.111 | 21.405 | 24.110 | 27.576 |
| 14.605 | 9.404 | 15.111 |  | 19.769 |
| 19.704 |  | 19.562 |  | 17.666 |
| 19.130 | 12.258 | 7.901 |  |  |
| 15.314 |  |  |  |  |

| Table Analyzed | Distance |  |  |  |  |
| --- | --- | --- | --- | --- | --- |
|  |  |  |  |  |  |
| ANOVA summary |  |  |  |  |  |
| F | 0.3903 |  |  |  |  |
| P value | 0.8147 |  |  |  |  |
| P value summary | ns |  |  |  |  |
| Are differences among means statistically significant? (P < 0.05) | No |  |  |  |  |
| R square | 0.02712 |  |  |  |  |
|  |  |  |  |  |  |
| Brown-Forsythe test |  |  |  |  |  |
| F (DFn. DFd) | 0.9425 (4. 56) |  |  |  |  |
| P value | 0.4463 |  |  |  |  |
| P value summary | ns |  |  |  |  |
| Significantly different standard deviations? (P < 0.05) | No |  |  |  |  |
|  |  |  |  |  |  |
| Bartlett's test |  |  |  |  |  |
| Bartlett's statistic (corrected) | 4.708 |  |  |  |  |
| P value | 0.3185 |  |  |  |  |
| P value summary | ns |  |  |  |  |
| Significantly different standard deviations? (P < 0.05) | No |  |  |  |  |
|  |  |  |  |  |  |
| ANOVA table | SS | DF | MS | F (DFn. DFd) | P value |
| Treatment (between columns) | 45.61 | 4 | 11.40 | F (4. 56) = 0.3903 | P = 0.8147 |
| Residual (within columns) | 1636 | 56 | 29.22 |  |  |
| Total | 1682 | 60 |  |  |  |

Data and statistics of figure 3 (B) – Rotations (number of rotations per 6 minutes)

| 6X | 4X | 2X | 1X | 1X/2D |
| --- | --- | --- | --- | --- |
| 19 | 21 | 19 | 25 | 23 |
| 10 | 15 | 29 | 31 | 28 |
| 10 | 6 | 22 | 14 | 14 |
| 21 | 18 | 8 | 10 | 9 |
| 6 | 16 | 10 | 19 | 24 |
| 16 | 12 | 25 | 17 | 14 |
| 30 | 18 | 19 | 18 | 27 |
| 15 | 19 | 15 | 23 | 9 |
| 10 |  | 13 | 6 | 12 |
| 6 | 17 | 23 | 19 | 34 |
| 26 | 11 | 21 | 5 | 17 |
| 7 | 10 | 5 |  | 18 |
| 16 | 10 | 10 |  |  |
| 24 |  |  |  |  |

| Table Analyzed | rotations |  |  |  |  |
| --- | --- | --- | --- | --- | --- |
|  |  |  |  |  |  |
| ANOVA summary |  |  |  |  |  |
| F | 0.7282 |  |  |  |  |
| P value | 0.5764 |  |  |  |  |
| P value summary | ns |  |  |  |  |
| Are differences among means statistically significant? (P < 0.05) | No |  |  |  |  |
| R square | 0.04862 |  |  |  |  |
|  |  |  |  |  |  |
| Brown-Forsythe test |  |  |  |  |  |
| F (DFn. DFd) | 0.8694 (4. 57) |  |  |  |  |
| P value | 0.4880 |  |  |  |  |
| P value summary | ns |  |  |  |  |
| Significantly different standard deviations? (P < 0.05) | No |  |  |  |  |
|  |  |  |  |  |  |
| Bartlett's test |  |  |  |  |  |
| Bartlett's statistic (corrected) | 4.016 |  |  |  |  |
| P value | 0.4039 |  |  |  |  |
| P value summary | ns |  |  |  |  |
| Significantly different standard deviations? (P < 0.05) | No |  |  |  |  |
|  |  |  |  |  |  |
| ANOVA table | SS | DF | MS | F (DFn. DFd) | P value |
| Treatment (between columns) | 152.5 | 4 | 38.13 | F (4. 57) = 0.7282 | P = 0.5764 |
| Residual (within columns) | 2985 | 57 | 52.37 |  |  |
| Total | 3137 | 61 |  |  |  |

Data and statistics of figure 3 (C) – time spent at the top zone (s).

| 6X | 4X | 2X | 1X | 1X/2D |
| --- | --- | --- | --- | --- |
| 32.4 | 24.8 | 25.5 | 8.0 | 0.1 |
|  | 34.3 | 58.5 | 53.1 | 28.0 |
| 47.4 |  | 0.8 | 5.6 | 86.1 |
| 23.3 | 9.1 | 0.1 | 1.4 | 106.2 |
| 24.0 | 16.0 | 96.4 | 4.0 | 49.1 |
| 20.4 | 26.1 | 53.5 | 0.1 | 65.6 |
| 34.3 | 34.7 | 16.4 | 15.3 | 71.8 |
| 16.7 | 27.9 | 8.3 | 9.1 | 9.7 |
| 0.1 | 0.1 |  |  | 106.0 |
| 4.4 | 11.4 | 59.5 | 6.4 | 44.3 |
| 8.6 | 39.6 | 32.9 | 1.5 | 43.7 |
| 9.1 |  | 60.5 |  | 16.1 |
| 2.4 | 42.4 |  |  |  |
| 3.4 |  |  |  |  |

| Table Analyzed | time top |
| --- | --- |
|  |  |
| Kruskal-Wallis test |  |
| P value | 0.0055 |
| Exact or approximate P value? | Approximate |
| P value summary | ** |
| Do the medians vary signif. (P < 0.05) | Yes |
| Number of groups | 5 |
| Kruskal-Wallis statistic | 14.65 |
|  |  |
| Data summary |  |
| Number of treatments (columns) | 5 |
| Number of values (total) | 57 |

| Number of families | 1 |  |  |
| --- | --- | --- | --- |
| Number of comparisons per family | 4 |  |  |
| Alpha | 0.05 |  |  |
|  |  |  |  |
| Dunn's multiple comparisons test | Mean rank diff. | Significant? | Summary |
|  |  |  |  |
| 2X vs. 6X | 10.56 | No | ns |
| 2X vs. 4X | 4.136 | No | ns |
| 2X vs. 1X | 17.91 | Yes | ** |
| 2X vs. 1X/2D | -6.841 | No | ns |

Data and statistics of figure 3 (D) – latency to the first entry in the top zone (s)

| 6X | 4X | 2X | 1X | 1X/2D |
| --- | --- | --- | --- | --- |
| 8.0 | 0.1 | 68.7 | 66.6 |  |
| 48.5 | 73.8 | 4.3 | 98.9 | 155.2 |
| 19.0 | 4.6 |  | 311.6 | 8.5 |
| 151.9 | 173.1 |  | 192.3 | 16.0 |
| 17.9 | 10.1 | 97.6 | 339.6 | 1.2 |
| 177.5 | 93.2 | 13.0 |  | 20.1 |
| 143.0 | 132.0 | 100.8 | 234.8 | 6.2 |
| 156.8 | 198.8 | 135.2 | 122.1 | 125.8 |
|  |  | 0.0 | 7.2 | 9.9 |
| 299.0 | 178.6 | 4.9 | 330.3 | 98.8 |
| 106.6 | 259.6 | 28.2 | 317.3 |  |
| 141.6 | 60.3 | 38.5 |  | 14.9 |
| 284.8 | 13.0 |  |  |  |
| 169.6 |  |  |  |  |

| Table Analyzed | latency to top |
| --- | --- |
|  |  |
| Kruskal-Wallis test |  |
| P value | 0.0053 |
| Exact or approximate P value? | Approximate |
| P value summary | ** |
| Do the medians vary signif. (P < 0.05) | Yes |
| Number of groups | 5 |
| Kruskal-Wallis statistic | 14.72 |
|  |  |
| Data summary |  |
| Number of treatments (columns) | 5 |
| Number of values (total) | 55 |

| Number of families | 1 |  |  |
| --- | --- | --- | --- |
| Number of comparisons per family | 4 |  |  |
| Alpha | 0.05 |  |  |
|  |  |  |  |
| Dunn's multiple comparisons test | Mean rank diff. | Significant? | Summary |
|  |  |  |  |
| 2X vs. 6X | -15.30 | No | ns |
| 2X vs. 4X | -8.742 | No | ns |
| 2X vs. 1X | -21.75 | Yes | ** |
| 2X vs. 1X/2D | 0.1500 | No | ns |

Data and statistics of figure 3 (E) – time spent at the tank bottom (s)

| 6X | 4X | 2X | 1X | 1X/2D |
| --- | --- | --- | --- | --- |
| 246.7 | 290.1 | 209.7 | 271.8 | 338.0 |
| 162.6 | 199.8 | 214.2 | 203.6 | 67.6 |
| 190.1 | 82.9 | 321.0 | 319.9 | 164.9 |
| 278.8 | 317.3 | 356.4 | 325.8 | 132.3 |
| 264.4 | 315.0 | 125.1 | 341.5 | 124.1 |
| 252.0 | 270.2 | 201.5 | 358.3 | 236.5 |
| 265.6 | 241.3 | 167.1 | 313.7 | 181.0 |
| 287.2 | 297.1 | 291.8 | 330.0 | 269.8 |
| 359.7 | 354.7 | 45.8 |  | 124.5 |
| 332.7 | 237.9 | 149.2 | 329.2 | 253.1 |
| 327.5 | 270.9 | 170.0 | 325.3 | 286.1 |
| 217.8 |  | 106.4 |  | 274.0 |
| 348.7 | 231.7 | 320.3 |  |  |
| 316.0 |  |  |  |  |

| Table Analyzed | | | time bottom | |  | |  | |  |  |
| --- | --- | --- | --- | --- | --- | --- | --- | --- | --- | --- |
|  | | |  | |  | |  | |  |  |
| ANOVA summary | | |  | |  | |  | |  |  |
| F | | | 4.621 | |  | |  | |  |  |
| P value | | | 0.0027 | |  | |  | |  |  |
| P value summary | | | ** | |  | |  | |  |  |
| Are differences among means statistically significant? (P < 0.05) | | | Yes | |  | |  | |  |  |
| R square | | | 0.2482 | |  | |  | |  |  |
|  | | |  | |  | |  | |  |  |
| Brown-Forsythe test | | |  | |  | |  | |  |  |
| F (DFn. DFd) | | | 2.408 (4. 56) | |  | |  | |  |  |
| P value | | | 0.0600 | |  | |  | |  |  |
| P value summary | | | ns | |  | |  | |  |  |
| Significantly different standard deviations? (P < 0.05) | | | No | |  | |  | |  |  |
|  | | |  | |  | |  | |  |  |
| Bartlett's test | | |  | |  | |  | |  |  |
| Bartlett's statistic (corrected) | | | 6.334 | |  | |  | |  |  |
| P value | | | 0.1755 | |  | |  | |  |  |
| P value summary | | | ns | |  | |  | |  |  |
| Significantly different standard deviations? (P < 0.05) | | | No | |  | |  | |  |  |
|  | | |  | |  | |  | |  |  |
| ANOVA table | | | SS | | DF | | MS | | F (DFn. DFd) | P value |
| Treatment (between columns) | | | 98187 | | 4 | | 24547 | | F (4. 56) = 4.621 | P = 0.0027 |
| Residual (within columns) | | | 297448 | | 56 | | 5312 | |  |  |
| Total | | | 395635 | | 60 | |  | |  |  |
| Number of families | 1 |  | |  | |  | |  |  |  |
| Number of comparisons per family | 4 |  | |  | |  | |  |  |  |
| Alpha | 0.05 |  | |  | |  | |  |  |  |
|  |  |  | |  | |  | |  |  |  |
| Dunnett's multiple comparisons test | Mean Diff. | 95% CI of diff. | | Significant? | | Summary | |  |  |  |
|  |  |  | |  | |  | |  |  |  |
| 2X vs. 6X | -68.95 | -139.5 to 1.608 | | No | | ns | |  |  |  |
| 2X vs. 4X | -53.04 | -126.4 to 20.29 | | No | | ns | |  |  |  |
| 2X vs. 1X | -105.9 | -182.9 to -28.82 | | Yes | | ** | |  |  |  |
| 2X vs. 1X/2D | 1.713 | -71.62 to 75.04 | | No | | ns | |  |  |  |

**FIGURE 4**

Data and statistics of figure 4 (A) - Whole-body glucose (mg/g tissue)

| 6X | 4X | 2X | 1X | 1X/2d |
| --- | --- | --- | --- | --- |
| 40.97117 | 34.23019 | 36.46154 | 34.54545 | 60.44 |
| 35.50835 | 65.91928 | 88.76923 | 36.96970 | 59.56 |
| 68.74052 | 40.95665 | 71.84615 | 31.36364 | 58.24 |
| 47.34446 | 74.73842 | 54.92308 | 30.90909 | 44.26 |
| 49.77238 | 29.59641 | 44.46154 | 44.54545 | 58.24 |
| 49.01366 | 66.81614 | 91.23077 | 46.96970 | 40.15 |
| 45.67527 | 54.40957 | 128.30770 | 43.18182 | 58.82 |
| 35.81184 | 114.05080 | 52.76923 | 41.36364 | 63.24 |
| 31.86646 | 63.67713 | 79.07692 | 40.75758 | 54.41 |
| 42.18513 | 100.14950 | 128.30770 | 57.12121 | 54.71 |
| 44.61305 | 57.54858 | 106.30770 | 46.66667 | 50.00 |
| 29.74203 | 45.88939 | 56.46154 | 34.54545 | 48.09 |
| 56.44917 | 62.33184 | 53.23077 | 40.30303 | 33.24 |

| Table Analyzed | Glucose |
| --- | --- |
|  |  |
| Kruskal-Wallis test |  |
| P value | 0.0003 |
| Exact or approximate P value? | Approximate |
| P value summary | *** |
| Do the medians vary signif. (P < 0.05) | Yes |
| Number of groups | 5 |
| Kruskal-Wallis statistic | 20.97 |
|  |  |
| Data summary |  |
| Number of treatments (columns) | 5 |
| Number of values (total) | 65 |

| Number of families | 1 |  |  |
| --- | --- | --- | --- |
| Number of comparisons per family | 4 |  |  |
| Alpha | 0.05 |  |  |
|  |  |  |  |
| Dunn's multiple comparisons test | Mean rank diff. | Significant? | Summary |
|  |  |  |  |
| 2X vs. 6X | 23.38 | Yes | ** |
| 2X vs. 4X | 6.846 | No | ns |
| 2X vs. 1X | 29.23 | Yes | *** |
| 2X vs. 1X/2d | 10.92 | No | ns |
|  |  |  |  |

Data and statistics of figure 4 (B) - Whole-body glycogen (mmol glucose/g tissue)

| 6X | 4X | 2X | 1X | 1X/2d |
| --- | --- | --- | --- | --- |
| 14.15297 | 7.198545 | 5.646731 | 7.980051 | 11.765510 |
| 11.98765 | 12.111400 | 14.297830 | 6.789026 | 13.639270 |
| 21.95816 | 8.043259 | 17.352970 | 9.660747 | 10.672540 |
| 15.60055 | 13.354570 | 11.969590 | 7.295163 | 8.841547 |
| 16.53094 | 8.138077 | 9.226937 | 9.552740 | 12.930070 |
| 15.99533 | 15.317380 | 15.797440 | 13.691410 | 9.935254 |
| 14.52198 | 10.388500 | 22.302960 | 9.851476 | 15.636380 |
| 11.41607 | 20.551470 | 13.064830 | 8.896744 | 14.435560 |
| 10.68840 | 13.025580 | 13.064050 | 10.443170 | 10.321800 |
| 14.40287 | 19.024720 | 27.225290 | 12.310350 | 12.712830 |
| 15.00081 | 10.953180 | 20.470170 | 11.162190 | 12.092610 |
| 10.05657 | 8.494502 | 9.808093 | 9.707755 | 9.956748 |
| 19.74449 |  | 8.257876 | 10.927180 | 12.352660 |

| Table Analyzed | Glycogen |
| --- | --- |
|  |  |
| Kruskal-Wallis test |  |
| P value | 0.0057 |
| Exact or approximate P value? | Approximate |
| P value summary | ** |
| Do the medians vary signif. (P < 0.05) | Yes |
| Number of groups | 5 |
| Kruskal-Wallis statistic | 14.58 |
|  |  |
| Data summary |  |
| Number of treatments (columns) | 5 |
| Number of values (total) | 64 |

| Number of families | 1 |  |  |
| --- | --- | --- | --- |
| Number of comparisons per family | 4 |  |  |
| Alpha | 0.05 |  |  |
|  |  |  |  |
| Dunn's multiple comparisons test | Mean rank diff. | Significant? | Summary |
|  |  |  |  |
| 2X vs. 6X | -6.769 | No | ns |
| 2X vs. 4X | 8.006 | No | ns |
| 2X vs. 1X | 19.69 | Yes | * |
| 2X vs. 1X/2d | 6.385 | No | ns |

Data and statistics of figure 4 (C) - Whole-body lactate (mmol/g tissue)

| 6X | 4X | 2X | 1X | 1X/2d |
| --- | --- | --- | --- | --- |
| 2.469750 | 3.018247 | 2.660589 | 3.70 | 3.415385 |
| 2.761125 | 3.478927 | 3.217721 | 3.38 | 4.098462 |
| 2.670938 | 4.209660 | 2.524149 | 3.36 | 4.146118 |
| 2.664000 | 4.289088 | 2.905045 | 3.27 | 4.154061 |
| 2.608500 | 3.852236 | 2.694699 | 3.87 | 3.550411 |
| 2.636250 | 3.463041 | 2.609424 | 3.51 | 3.717209 |
| 2.643188 | 3.423327 | 2.779974 | 3.68 | 4.273202 |
| 2.407313 | 3.081789 | 2.825455 | 3.22 | 4.233488 |
| 2.726438 | 3.002361 | 2.791344 | 3.50 | 3.963435 |
| 2.670938 | 3.328014 | 2.859565 | 3.23 | 4.098462 |
| 2.761125 | 3.812522 | 2.814085 | 3.72 | 4.042862 |
| 2.740313 | 3.002361 | 3.030115 | 3.48 | 4.400286 |
| 2.677875 |  | 2.461613 | 3.72 | 3.804580 |

| Table Analyzed | lactate |
| --- | --- |
|  |  |
| Kruskal-Wallis test |  |
| P value | < 0.0001 |
| Exact or approximate P value? | Approximate |
| P value summary | **** |
| Do the medians vary signif. (P < 0.05) | Yes |
| Number of groups | 5 |
| Kruskal-Wallis statistic | 49.61 |
|  |  |
| Data summary |  |
| Number of treatments (columns) | 5 |
| Number of values (total) | 64 |

| Number of families | 1 |  |  |
| --- | --- | --- | --- |
| Number of comparisons per family | 4 |  |  |
| Alpha | 0.05 |  |  |
|  |  |  |  |
| Dunn's multiple comparisons test | Mean rank diff. | Significant? | Summary |
|  |  |  |  |
| 2X vs. 6X | 6.615 | No | ns |
| 2X vs. 4X | -23.26 | Yes | ** |
| 2X vs. 1X | -24.00 | Yes | ** |
| 2X vs. 1X/2d | -37.08 | Yes | **** |

Weight of fish

Weight of fish used in the 1^st^ experiment

|  | Group 1 – 30 min | Group 2 – 3h | Group 3 – 6h | Group 4 – 12h | Group 5 – 24h | Group 6 – 48h |
| --- | --- | --- | --- | --- | --- | --- |
|  | 0.47 | 0.39 | 0.33 | 0.66 | 0.7 | 0.6 |
|  | 0.45 | 0.54 | 0.54 | 0.45 | 0.71 | 0.7 |
|  | 0.54 | 0.39 | 0.38 | 0.45 | 0.39 | 0.54 |
|  | 0.66 | 0.45 | 0.45 | 0.51 | 0.34 | 0.61 |
|  | 0.44 | 0.33 | 0.44 | 0.53 | 0.44 | 0.45 |
|  | 0.39 | 0.37 | 0.49 | 0.6 | 0.66 | 0.48 |
|  | 0.45 | 0.45 | 0.66 | 0.45 | 0.57 | 0.53 |
|  | 0.54 | 0.39 | 0.62 | 0.59 | 0.54 | 0.39 |
|  | 0.71 | 0.66 | 0.45 | 0.57 | 0.55 | 0.4 |
|  | 0.5 | 0.41 | 0.36 | 0.39 | 0.54 | 0.55 |
|  | 0.45 | 0.45 | 0.39 | 0.33 | 0.45 | 0.45 |
|  | 0.55 | 0.44 | 0.47 | 0.54 | 0.6 | 0.6 |
| Mean | 0.5125 | 0.439167 | 0.465 | 0.505833 | 0.540833 | 0.525 |
| SEM | 0.027195 | 0.02524 | 0.029064 | 0.027426 | 0.034167 | 0.027122 |

| ANOVA summary |  |  |  |  |  |
| --- | --- | --- | --- | --- | --- |
| F | 1,817 |  |  |  |  |
| P value | 0,1215 |  |  |  |  |
| P value summary | ns |  |  |  |  |
| Are differences among means statistically significant? (P < 0.05) | No |  |  |  |  |
| R square | 0,1210 |  |  |  |  |
|  |  |  |  |  |  |
| Brown-Forsythe test |  |  |  |  |  |
| F (DFn, DFd) | 0,3163 (5, 66) |  |  |  |  |
| P value | 0,9015 |  |  |  |  |
| P value summary | ns |  |  |  |  |
| Significantly different standard deviations? (P < 0.05) | No |  |  |  |  |
|  |  |  |  |  |  |
| Bartlett's test |  |  |  |  |  |
| Bartlett's statistic (corrected) | 1,216 |  |  |  |  |
| P value | 0,9434 |  |  |  |  |
| P value summary | ns |  |  |  |  |
| Significantly different standard deviations? (P < 0.05) | No |  |  |  |  |
|  |  |  |  |  |  |
| ANOVA table | SS | DF | MS | F (DFn, DFd) | P value |
| Treatment (between columns) | 0,08863 | 5 | 0,01773 | F (5, 66) = 1,817 | P = 0,1215 |
| Residual (within columns) | 0,6437 | 66 | 0,009753 |  |  |
| Total | 0,7323 | 71 |  |  |  |

Initial and final weight of fish used in the 2^nd^ experiment

|  | Group 1 6x | | Group 2 4x | | Group 3 2x | | Group 4 1x | | Group 5 1x/2d | |
| --- | --- | --- | --- | --- | --- | --- | --- | --- | --- | --- |
|  | Initial | Final | Initial | Final | Initial | Final | Initial | Final | Initial | Final |
|  | 0.34 | 0.41 | 0.39 | 0.42 | 0.59 | 0.82 | 0.59 | 0.64 | 0.46 | 0.53 |
|  | 0.47 | 0.56 | 0.81 | 1.05 | 0.38 | 0.46 | 0.55 | 0.56 | 0.36 | 0.41 |
|  | 0.55 | 0.65 | 0.59 | 0.77 | 0.33 | 0.39 | 0.52 | 0.6 | 0.52 | 0.57 |
|  | 0.4 | 0.44 | 0.47 | 0.52 | 0.63 | 0.66 | 0.71 | 0.79 | 0.37 | 0.41 |
|  | 0.34 | 0.37 | 0.55 | 0.56 | 0.45 | 0.51 | 0.47 | 0.56 | 0.41 | 0.4 |
|  | 0.62 | 0.56 | 0.47 | 0.59 | 0.33 | 0.32 | 0.41 | 0.44 | 0.41 | 0.43 |
|  | 0.57 | 0.67 | 0.49 | 0.54 | 0.61 | 0.84 | 0.37 | 0.42 | 0.46 | 0.48 |
|  | 0.61 | 0.68 | 0.47 | 0.68 | 0.37 | - | 0.48 | 0.48 | 0.56 | 0.59 |
|  | 0.44 | 0.46 | 0.44 | 0.48 | 0.4 | 0.44 | 0.44 | 0.47 | 0.37 | 0.36 |
|  | 0.7 | 0.74 | 0.5 | 0.57 | 0.57 | 0.67 | 0.35 | 0.39 | 0.62 | 0.61 |
|  | 0.36 | 0.45 | 0.43 | 0.49 | 0.47 | 0.55 | 0.63 | 0.67 | 0.46 | 0.49 |
|  | 0.54 | 0.63 | 0.41 | 0.45 | 0.47 | 0.54 | 0.38 | 0.45 | 0.52 | 0.51 |
|  | 0.39 | 0.41 | 0.66 | 0.73 | 0.74 | 1.05 | 0.7 | 0.82 | 0.69 | 0.72 |
|  | 0.48 | 0.5 | 0.32 | 0.42 | 0.42 | 0.63 | 0.52 | 0.56 | 0.47 | 0.51 |
| Mean | 0.495 | 0.551667 | 0.501667 | 0.593333 | 0.466667 | 0.563636 | 0.491667 | 0.539167 | 0.46 | 0.4825 |
| SEM | 0.033502 | 0.034089 | 0.031084 | 0.048131 | 0.03039 | 0.046564 | 0.031062 | 0.033166 | 0.022749 | 0.022691 |

Comparison of the initial weight among treatments:

| ANOVA summary |  |  |  |  |  |
| --- | --- | --- | --- | --- | --- |
| F | 0,3780 |  |  |  |  |
| P value | 0,8235 |  |  |  |  |
| P value summary | ns |  |  |  |  |
| Are differences among means statistically significant? (P < 0.05) | No |  |  |  |  |
| R square | 0,02458 |  |  |  |  |
|  |  |  |  |  |  |
| Brown-Forsythe test |  |  |  |  |  |
| F (DFn, DFd) | 0,6539 (4, 60) |  |  |  |  |
| P value | 0,6264 |  |  |  |  |
| P value summary | ns |  |  |  |  |
| Significantly different standard deviations? (P < 0.05) | No |  |  |  |  |
|  |  |  |  |  |  |
| Bartlett's test |  |  |  |  |  |
| Bartlett's statistic (corrected) | 4,707 |  |  |  |  |
| P value | 0,3186 |  |  |  |  |
| P value summary | ns |  |  |  |  |
| Significantly different standard deviations? (P < 0.05) | No |  |  |  |  |
|  |  |  |  |  |  |
| ANOVA table | SS | DF | MS | F (DFn, DFd) | P value |
| Treatment (between columns) | 399,5 | 4 | 99,87 | F (4, 60) = 0,3780 | P = 0,8235 |
| Residual (within columns) | 15853 | 60 | 264,2 |  |  |
| Total | 16253 | 64 |  |  |  |

Comparison of the final weight among treatments:

| ANOVA summary |  |  |  |  |  |
| --- | --- | --- | --- | --- | --- |
| F | 1,329 |  |  |  |  |
| P value | 0,2698 |  |  |  |  |
| P value summary | ns |  |  |  |  |
| Are differences among means statistically significant? (P < 0.05) | No |  |  |  |  |
| R square | 0,08266 |  |  |  |  |
|  |  |  |  |  |  |
| Brown-Forsythe test |  |  |  |  |  |
| F (DFn, DFd) | 0,4678 (4, 59) |  |  |  |  |
| P value | 0,7591 |  |  |  |  |
| P value summary | ns |  |  |  |  |
| Significantly different standard deviations? (P < 0.05) | No |  |  |  |  |
|  |  |  |  |  |  |
| Bartlett's test |  |  |  |  |  |
| Bartlett's statistic (corrected) | 3,578 |  |  |  |  |
| P value | 0,4662 |  |  |  |  |
| P value summary | ns |  |  |  |  |
| Significantly different standard deviations? (P < 0.05) | No |  |  |  |  |
|  |  |  |  |  |  |
| ANOVA table | SS | DF | MS | F (DFn, DFd) | P value |
| Treatment (between columns) | 1628 | 4 | 407,0 | F (4, 59) = 1,329 | P = 0,2698 |
| Residual (within columns) | 18067 | 59 | 306,2 |  |  |
| Total | 19694 | 63 |  |  |  |

Comparisons of initial and final weight in each treatment

Group 1, 6x

| Unpaired t test |  |
| --- | --- |
| P value | 0,0469 |
| P value summary | * |
| Significantly different? (P < 0.05) | Yes |
| One- or two-tailed P value? | Two-tailed |
| t, df | t=2,095 df=24 |

Group 2, 4x

| Unpaired t test |  |
| --- | --- |
| P value | 0,0769 |
| P value summary | ns |
| Significantly different? (P < 0.05) | No |
| One- or two-tailed P value? | Two-tailed |
| t, df | t=1,848 df=24 |

Group 3, 2x

| Unpaired t test |  |
| --- | --- |
| P value | 0,0789 |
| P value summary | ns |
| Significantly different? (P < 0.05) | No |
| One- or two-tailed P value? | Two-tailed |
| t, df | t=1,839 df=23 |

Group 4, 1x

| Unpaired t test |  |
| --- | --- |
| P value | 0,3982 |
| P value summary | ns |
| Significantly different? (P < 0.05) | No |
| One- or two-tailed P value? | Two-tailed |
| t, df | t=0,8602 df=24 |

Group 5, 1x/2day

| Unpaired t test |  |
| --- | --- |
| P value | 0,9315 |
| P value summary | ns |
| Significantly different? (P < 0.05) | No |
| One- or two-tailed P value? | Two-tailed |
| t, df | t=0,08684 df=24 |

Comparisons between growths among treatments.

|  | Group 1 6x | Group 2 4x | Group 3 2x | | Group 4 1x | Group 5 1x/2d | |
| --- | --- | --- | --- | --- | --- | --- | --- |
|  | 0.07 | 0.03 | 0.23 | | 0.05 | 0.07 | |
|  | 0.09 | 0.24 | 0.08 | | 0.01 | 0.05 | |
|  | 0.1 | 0.18 | 0.06 | | 0.08 | 0.05 | |
|  | 0.04 | 0.05 | 0.03 | | 0.08 | 0.04 | |
|  | 0.03 | 0.01 | 0.06 | | 0.09 | -0.01 | |
|  | -0.06 | 0.12 | -0.01 | | 0.03 | 0.02 | |
|  | 0.1 | 0.05 | 0.23 | | 0.05 | 0.02 | |
|  | 0.07 | 0.21 | - | | 0 | 0.03 | |
|  | 0.02 | 0.04 | 0.04 | | 0.03 | -0.01 | |
|  | 0.04 | 0.07 | 0.1 | | 0.04 | -0.01 | |
|  | 0.09 | 0.06 | 0.08 | | 0.04 | 0.03 | |
|  | 0.09 | 0.04 | 0.07 | | 0.07 | -0.01 | |
|  | 0.02 | 0.07 | 0.31 | | 0.12 | 0.03 | |
|  | 0.02 | 0.1 | 0.21 | | 0.04 | 0.04 | |
| Mean | 0.051 | 0.091 | 0.115 | | 0.052 | 0.024 | |
| SD | 0.045 | 0.071 | 0.097 | | 0.033 | 0.026 | |
| Kruskal-Wallis test | | | |  | | |  |
| P value | | | | 0,0042 | | |  |
| Exact or approximate P value? | | | | Approximate | | |  |
| P value summary | | | | ** | | |  |
| Do the medians vary signif. (P < 0.05) | | | | Yes | | |  |
| Number of groups | | | | 5 | | |  |
| Kruskal-Wallis statistic | | | | 15,24 | | |  |
|  | | | |  | | |  |
| Data summary | | | |  | | |  |
| Number of treatments (columns) | | | | 5 | | |  |
| Number of values (total) | | | | 69 | | |  |

| Number of families | 1 |  |  |  |  |
| --- | --- | --- | --- | --- | --- |
| Number of comparisons per family | 4 |  |  |  |  |
| Alpha | 0,05 |  |  |  |  |
|  |  |  |  |  |  |
| Dunn's multiple comparisons test | Mean rank diff, | Significant? | Summary |  |  |
|  |  |  |  |  |  |
| Group 3 2x vs. Group 1 6x | 11,87 | No | ns |  |  |
| Group 3 2x vs. Group 2 4x | 3,481 | No | ns |  |  |
| Group 3 2x vs. Group 4 1x | 12,80 | No | ns |  |  |
| Group 3 2x vs. Group 5 1x/2d | 27,20 | Yes | ** |  |  |
|  |  |  |  |  |  |
|  |  |  |  |  |  |
| Test details | Mean rank 1 | Mean rank 2 | Mean rank diff, | n1 | n2 |
|  |  |  |  |  |  |
| Group 3 2x vs. Group 1 6x | 46,23 | 34,36 | 11,87 | 13 | 14 |
| Group 3 2x vs. Group 2 4x | 46,23 | 42,75 | 3,481 | 13 | 14 |
| Group 3 2x vs. Group 4 1x | 46,23 | 33,43 | 12,80 | 13 | 14 |
| Group 3 2x vs. Group 5 1x/2d | 46,23 | 19,04 | 27,20 | 13 | 14 |
